# A Study on Risk Factors of Cardiovascular Diseases in an Urban Health Center of Kolkata

**DOI:** 10.4103/0970-0218.43239

**Published:** 2008-10

**Authors:** Soumya Deb, Aparajita Dasgupta

**Affiliations:** Department of Preventive and Social Medicine, All India Institute of Hygiene and Public Health, 110 C.R. Avenue, Kolkata - 700 073, India

## Introduction

Cardiovascular disease is the world's leading killer accounting for 16.7 million deaths or 29.2% of the total number of global deaths in 2003. While deaths from heart attacks have declined more than 50% since the 1960s in many industrialized countries, 80% of global cardiovascular–disease-related deaths now occur in low- and middle-income nations, which cover most countries in Asia.(1)

In India in the past five decades, rates of coronary disease among urban populations have risen from 4% to 11%. The World Health Organization (WHO) estimates that 60% of the world's cardiac patients will be Indian by 2010. Nearly 50% of cardiovascular-related deaths in India occur in patients below the age of 70, compared with just 22% in the West. This trend is particularly alarming because of its potential impact on one of Asia's fastest-growing economies.(2)

In India, the leading cause of death is cardiovascular disease. At the same time, it has been found that cardiovascular disease is third overall in the burden of disease, the other two being infectious and parasitic diseases and unintentional injuries. In India, deaths from coronary heart disease rose from 1.17 million in 1990 to 1.59 million in 2000 and is expected to rise to 2.03 million in 2010. The prevalence varies by site, age group studied, and diagnostic criteria used, but an urban prevalence of about 10% in adults aged ≥35 years old is a credible estimate based on several surveys.(3)

With this in the backdrop, a descriptive, observational study with a cross-sectional study design was conducted to assess the prevalence of risk factors in patients > 30 years old attending the sector clinic in the Urban Health Centre, Chetla in the city of Kolkata. The health center functions under the auspices of the All India Institute of Hygiene and Public Health, Kolkata and caters to a huge slum population in the Chetla area. Residents of the urban slum attend the sector outdoors (OPD) 5 days every week (except Saturdays and Sundays) for different medical ailments. Two days of the week were randomly chosen (Wednesday and Friday) and the researchers included the patients >30 years old who attended the sector OPD during those two days in the study. The study was conducted for a period of 4 months during 2006 to 2007.

## Materials and Methods

The study aimed to assess the prevalence of different risk factors of cardiovascular disease in the study population and the association, if any, of these risk factors with their sociodemographic variables. All patients >30 years old attending the sector OPD on every Wednesday and Friday during the study period were included in the study. Overall, a total of 480 patients from the slum belonging to all age groups attended the sector OPD during the study period. Of those patients, 269 were more than 30 years old. Seven patients refused to participate in the study and 16 patients had documentary evidence of diagnosed cardiovascular disease. Thus, a total of 246 patients were included in the study. Patients not willing to participate and those with diagnosed cardiovascular disease (based on documentary evidence) were excluded. (The reason for excluding patients with diagnosed cardiovascular diseases was that they are likely to have received health education and advice from the doctor following their diagnosis of cardiovascular disease and might have changed their lifestyle, dietary habits, and physical activity practices, so an accurate depiction might not be reflected in the study if they were included).

Cardiovascular diseases encompassing coronary heart disease, angina pectoris, hypertension, myocardial infarction cerebrovascular disease (stroke), and congestive heart failure (excluding the organic and congenital cardiovascular disease) were considered in this study. Although hypertension itself is a cardiovascular disease, it is also one of the most important risk factors of other cardiovascular diseases. So, it has been included in the list of risk factors measured during the study.

The following tools were used during the study: a pre-designed, semi-structured questionnaire, a Mercury sphygmomanometer, measuring tape, and a weighing machine. Informed consent was obtained from each respondent prior to the interview and physical examination. The same instruments were used for measuring the different health parameters to maintain uniformity. Only proven risk factors as obtained by the review of literature were taken for the study.(3) The risk factors for physical activity and dietary pattern were taken from the standard Integrated Disease Surveillance Program questionnaire while the scoring for these risk factors was done by the researchers themselves. The prevalence of these risk factors and their association with different sociodemographic variables like age, sex, literacy status, and per capita monthly income were analyzed.

## Results and Discussion

The study population comprised of an equal number of males and females—123 each. Most of the males (17%) were between the ages of 40 and 49 years old while the maximum (19%) number of females were aged 60 years old and above. Overall, 75 patients (30%) belonged to the geriatric age group (≥60 years age). A total of 111 study participants (45%) were illiterate while only 29% had secondary education and above. Table 1 shows the scoring of general risk factors and its relation to sociodemographic variables of the study population. It was observed that all 12 participants who secured 0 (which means absence of risk factor) were below 50 years old. The maximum attainable score was 11 and a highest of 5 was obtained by two participants, both above 50 years old. The risk factor scores increased significantly with increase in age of the study population (*P*=0.0368). The score of ≤ 2 was obtained more among the 30–39 year old age group than the ≥60 year old age group (70% *vs.* 44%). The reverse was true for ≥3 scores in the above two age groups, respectively (30% *vs.* 56%). It was also observed that the risk factor score of ≥3 was significantly more in the higher income group that is ≥ Rs 500 monthly per capita income (PCI) group as compared with the <Rs 500 PCI group (55% *vs.* 29%) (*P*=0.0224). The risk factor score was also found to be higher in males as compared with females.

**Table 1:** General risk factor scores in relation to sociodemographic variables (n=246)

Risk factor score*	Age (in years)				
	
	30–39	40–49	50–59	60 and above	Total
0	6 (13)	6 (8)	-	-	12 (5)
1	12 (27)	6 (8)	3 (6)	6 (8)	27 (11)
2	18 (40)	36 (48)	15 (29)	27 (36)	96 (39)
3	6 (13)	18 (24)	9 (18)	21 (28)	54 (22)
4	3 (7)	9 (12)	21 (41)	18 (24)	51 (21)
5	-	-	3 (6)	3 (4)	6 (2)
Total	45 (100)	75 (100)	51 (100)	75 (100)	246 (100)
	(19)	(30)	(21)	(30)	(100)

Chi sq = 8.4; df = 3; *P* = 0.0368 (<0.05). Figures in parentheses are in percentages.

**Table:** 

Risk factor score	Male	Female
0	9 (7)	3 (2)
1	9 (7)	18 (15)
2	42 (34)	54 (44)
3	33 (27)	21 (17)
4	24 (20)	27 (22)
5	2 (5)	-
Total	123 (100)	123 (100)
	(50)	(50)

Chi sq = 1.23; df = 1; *P* = 0.2671. Figures in parentheses are in percentages.

**Table:** 

Risk factor score	PCI<Rs. 500	PCI≥Rs. 500
0	9 (10)	3 (2)
1	6 (6)	21 (14)
2	51 (55)	45 (29)
3	9 (10)	45 (29)
4	15 (16)	36 (24)
5	3 (3)	3 (2)
Total	93 (100)	153 (100)
	(38)	(62)

Chi sq = 5.21; df = 1; *P* = 0.0224 (<0.05); PCI = Per capita income.

* The lowest score obtained was 0 and the highest score obtained was 5. Figures in parentheses are in percentages.

Table 2 shows the relation between physical activity and dietary scores with various sociodemographic variables. The higher the score, the higher the risk and vice versa. The highest physical activity score obtained by the study population was 12 out of maximum possible score of 14. Ideally, a score of 0 signified a minimum risk for cardiovascular disease but unfortunately, the minimum score that could be attained by the study participants was 7. In the 30–39 year old group, only 27% of the study population practiced high-risk physical activity. On the other hand, in the geriatric age group (>60 yrs), a majority (83%) of the study population practiced a high-risk physical activity pattern (*P*=0.0028). Besides, a high-risk physical activity pattern was practiced more by the literate and higher income group as compared with the illiterate and the relatively lower income group (66% *vs.* 51% and 72% *vs.* 49%, respectively). The number of females practicing high-risk physical activity was also slightly more when compared with males (61% *vs.* 58%). With regard to the dietary score, the lowest score obtained by the study population was 1 while the highest score secured was 4 (the minimum and maximum obtainable scores were 0 and 4, respectively). Accordingly, a low-risk dietary pattern was considered for those obtaining a score of 1–2 while a high-risk dietary pattern was considered for those obtaining a score of 3–4. A high-risk dietary pattern was found to be significantly more in the literate group as compared with the illiterate group (40% *vs.* 19%, respectively and *P*=0.0309). It was also observed that the high-risk dietary pattern was practiced more by the younger age group as compared with the elders (*P*< 0.05). Besides, those with higher monthly PCI (≥Rs 500) were at increased risk with regard to dietary habit as compared with the lower income group (35% *vs.* 23%). With regard to individual risk factors in the study population, the prevalent risk factors such as a history of hypertension was found to be 28%, history of diabetes was 13%, significant family history was 17%, smoking was 21%, chewing non smoking tobacco was 18%, hypertension measured during the study was 46% (39% for Stage I hypertension and 7% for Stage II hypertension), and high body mass index (BMI) was 56%. A comparison with data published recently from a random sample population from Jaipur in north India revealed the following: the prevalence of smoking was higher in north India (37%) as compared with the present study (21%), the prevalence of hypertension in the present study was higher (46%) when compared with the north Indian study (37%), and the prevalence of diabetes was almost the same (12% in the north Indian study *vs.* 13% in the present study). Obesity was more prevalent in the current study (56%) when compared with the north Indian study (27%). Another cross-sectional study conducted on a study population aged 20–59 years old in an industrial population in Delhi,(4) when compared with the present study showed a prevalence of hypertension (30% *vs.* 46%), diabetes (15% *vs.* 13%), current smoking (36% *vs.* 21%), obesity (35% *vs.* 56%), and the presence of at least two risk factors (47% *vs.* 55%). Another study conducted in Andhra Pradesh(5) when compared with the present study showed a prevalence of diabetes (24% *vs.* 13%), hypertension (28% *vs.* 46%), smoking (24% *vs.* 21%), positive family history (14% *vs.* 17%), and obesity (36% *vs.* 56%). The regional differences in various parameters in the different studies are interesting.

**Table:** Scoring for general risk factors

General risk factor score	Scores	
Risk factor	Present	Absent
History of hypertension	1	0
History of diabetes	1	0
History of stroke	1	0
Family history of hypertension	1	0
Family history of diabetes	1	0
Regular smoker	1	0
Regular non smoking tobacco user	1	0
BMI>24.9	1	0
Stage I HTN (systolic 140–159 / diastolic 90–99)	1	0
Stage II HTN (systolic 160–179 / diastolic 100–109)	2	-
Stage III HTN (systolic ≥180 / diastolic ≥110)	3	-

Maximum attainable score = 11; minimum attainable score = 0; BMI = Body mass index; HTN = Hypertension

**Table:** Scoring for physical activity

Physical activity score	Score
Nature of work
Sedentary	2
Moderate	1
Heavy	0
Average number of hours spent at work/day
<5 hrs	2
5–10 hrs	1
>10 hrs	0
Mode of traveling for going to workplace
No travel	3
Sitting in vehicle/standing	2
Walking	1
Cycling	0
Physical activity not related to work
No such physical activity	5
Slow walking	4
Brisk walking	3
Yoga	2
Cycling	1
Physically active games	0

Maximum attainable score = 12; minimum attainable score = 0

**Table:** Scoring for dietary pattern

Dietary pattern score	Score
Number of days of vegetable intake/week
<4	1
≥4	0
Number of days of fruit intake/week
<4	1
≥4	0
Daily oil consumption/adult consumption unit
≥20 ml/day	1
<20 ml/day	0
Extra salt intake with cooked food
Yes	1
No	0

Maximum attainable score = 4; minimum attainable score = 0

**Table 2:** Physical activity and dietary risk factor score in relation to sociodemographic variables (n=246)

Age in years						

Physical activity score*	30–39 (n=45)	40–49 (n=75)	50–59 (n=51)	>=60 (n=75)	Total (n=246)	Chi. sq test
7–8 (low risk)	15 (33%)	18 (24%)	11 (22%)	4(5%)	48 (20%)	
9–10 (moderate risk)	18 (40%)	9 (12%)	15 (29%)	9 (12%)	51 (21%)	Chi sq = 11.75 df=2
11–12 (high risk)	12 (27%)	48 (64%)	25 (49%)	62 (83%)	147 (59%)	*P* = 0.0028 (<0.05)
Diet score**						
1–2 (low risk)	29 (64%)	44 (59%)	37 (73%)	61 (81%)	171 (70%)	Chi sq = 9.88 df=2
3–4 (high risk)	16 (36%)	31 (41%)	14 (27%)	14 (19%)	75 (30%)	*P* = 0.0196 (<0.05)

Physical activity score	Male (n=123)	Female (n=123)	Total (n=246)	Chi. sq test
7–8 (low risk)	22 (18%)	26 (21%)	48 (20%)	-
9–10 (moderate risk)	29 (24%)	22 (18%)	51 (21%)	Chi. sq = 1.36 df=2
11–12 (high risk)	72 (58%)	75 (61%)	147 (59%)	*P* = 0.507 (>0.05)
Diet score				
1–2 (low risk)	82 (67%)	89 (72%)	171 (70%)	Chi. sq = 0.94 df=2
3–4 (high risk)	41 (33%)	34 (28%)	75 (30%)	*P* = 0.332 (>0.05)

PCI (in Rs)				

Physical activity score <	500 (n=93)	≥500 (n=153)	Total (n=246)	Chi. Sq test
7–8 (low risk)	24 (26%)	24 (16%)	48 (20%)	
9–10 (moderate risk)	33 (35%)	18(12%)	51 (21%)	Chi. sq = 9.94 df=1
11–12 (high risk)	36 (49%)	111 (72%)	147 (59%)	*P* = 0.0069(>0.05)
Diet score				
1–2 (low risk)	72 (77%)	99(65%)	171 (70%)	Chi. sq = 1.47 df = 1
3–4 (high risk)	21 (23%)	54 (35%)	75 (30%)	*P* = 0.225 (>0.05)

Physical activity score	Illiterate (n=111)	Literate (n=135)	Total (n=246)	Chi. sq test
7–8 (low risk)	24 (22%)	24 (18%)	48 (20%)	
9–10 (moderate risk)	30 (27%)	21 (16%)	90 (66%)	Chi. sq = 9.94 df=1
11–12 (high risk)	48 (20%)	51 (21%)	147 (59%)	*P* = 0.0069(<0.05)
Diet score				
1–2 (low risk)	90 (81%)	81 (60%)	171 (70%)	Chi. sq = 1.47 df = 1
3–4 (high risk)	21 (19%)	54 (40%)	75 (30%)	*P* = 0.225 (>0.05)

* The lowest score obtained was 7 while the highest score obtained was 12.

** The lowest score obtained was 1 while the highest score obtained was 4.

## Conclusion

Although the absence of well-established disease surveillance mechanisms prevents a precise estimation of the size of cardiovascular disease burdens, the direction of change is clear—the burden is rising. Even with the current status of knowledge, however, the magnitude of the problem is large enough to demand urgent attention and action. This study revealed that the risk factors for cardiovascular disease were highly prevalent in the study population, the most striking being obesity (56%) and hypertension as per WHO criteria (46%). The high-risk physical activity score was significantly more in the younger age group and higher income group. A high-risk dietary pattern was seen to be significantly more in the younger age group and the literate group. All the respondents were given health education about ways of preventing cardiovascular diseases. Besides, a session was arranged by the researchers for the health workers to educate them about the various risk factors of cardiovascular diseases and ways to prevent them so that they can inculcate the preventive measures among the people living in the slums during their house-to-house visits. This gains importance especially since many of the risk factors like smoking, obesity, dietary pattern, physical inactivity, etc. are modifiable. Urgent measures based on primordial and primary prevention need to be taken especially from the school level to modify the lifestyle and behavior of the people of the slum community otherwise the epidemic of non communicable disease may get out of hand.
